# Design for a digital twin in clinical patient care

**DOI:** 10.1038/s44401-025-00060-1

**Published:** 2026-02-02

**Authors:** Anna-Katharina Nitschke, Carlos Brandl, Fabian Egersdörfer, Magdalena Görtz, Markus Hohenfellner, Matthias Weidemüller

**Affiliations:** 1 Physikalisches Institut, Universität Heidelberg, Heidelberg, Germany; 2 Junior Clinical Cooperation Unit ‘Multiparametric Methods for Early Detection of Prostate Cancer’, German Cancer Research Center (DKFZ), Heidelberg, Germany; 3 Department of Urology, Heidelberg University Hospital, Heidelberg, Germany

**Keywords:** Predictive medicine, Computational science

## Abstract

Digital Twins hold great potential to personalize clinical patient care, provided the concept is translated to meet specific requirements emerging from established clinical workflows. We present a general and unspecialized Digital Twin design combining knowledge graphs and ensemble learning to reflect the entire patient’s clinical journey and assist clinicians in their decision-making. Such a design is predictive, modular, evolving, informed, interpretable and explainable, thus opening broad clinical applications.

## Introduction

In the era of precision medicine, Digital Twins (DTs) are emerging as a long-term goal with the potential to revolutionize healthcare delivery and have experienced a surge of publications in recent years^1,2^. Precision medicine aims at leveraging multi-omic, demographic, environmental, and lifestyle patient data to improve the prediction of disease occurrences and deliver personalized treatment recommendations^3^. The DT as a detailed, digital replica of an individual patient extends the definition of precision medicine by enabling drug discovery and development, real-time health monitoring, and surgery planning and rehearsal^4,5^. For explicit clinical applications, DTs of organs, like the heart, have been proposed. Those are usually driven by mechanistic models, simulating variables like blood flow and blood pressure to create synthetic physiological data like photoplethysmograms^6^. Recent advances in machine learning have enabled well-performing models for many clinical tasks, which can form a basis for a predictive DT. The combination of mechanistic models with machine learning models is conceptually desired, but the integration of (multiple) machine learning and mechanistic models into a unified framework is still part of active research^7,8^.

A desired application for DTs in the field of personalized medicine is the representation of patients along their whole clinical journey. Commonly, DTs for personalized medicine are tailored to a specific disease or medical question (like examples summarized within Vallé’s viewpoint^9^). It is difficult to extend such DTs towards other diseases or new procedures. First approaches on how to cover clinical patient journeys by DT of patients have been presented, for example, by Voigt et al. for personalized multiple sclerosis care by outlining patient data streams, clinical workflows and procedures, existing models, prior knowledge, and open challenges^10^. One central problem for the development of DTs in precision medicine is the lack of uniform methods, standards and norms. There are different challenges associated with different stages of the development of a Digital Twin, including data collection, data storage, governance, algorithmic designs and human interaction^10^.

In the following, we focus on the derivation of a uniform design of a DT on the algorithmic level. De Domenico et al. argue that the complexity of disease and therefore personalized patient care requires modular, non-specialized DT designs which can effectively integrate multiple subsystems for specialized cases^11^. One example of an unspecialized modular DT design has been published by Gaebel et al., using a knowledge graph-based framework^12^. Such knowledge graph-based frameworks can describe and connect specialized data processing models and their causal relationships, e.g., by Resource Description Frameworks (RDFs)^12^. One disease-specific implementation of a knowledge graph-based DT was performed by Grieb et al.^13^ for multiple myeloma treatment. They implemented a recursive model structure and described implementation strategies on a technical level. They highlighted the benefits of using knowledge graphs, such as the integration of multiple heterogeneous data sources and specialised modules, as well as the inclusion of medical knowledge and guidelines. However, Gaebel et al. acknowledge that one difficulty of their design lies within the lack of a strategy for managing redundancies, which emerge from the fragmented clinical domains, where many models might provide estimates of the same information^12^.

This perspective article presents a general and unspecialized design for a patient DT to reflect the evolving clinical patient journey based on a bipartite knowledge graph, combining patient attributes derived from patient data and preexisting predictive models. We designed an overhead algorithm to enable a meaningful simulation of the patient journey. The algorithm orchestrates the execution of several preexisting specialized models with similar tasks, as well as with different tasks. The central part of the DT’s model orchestration is the introduction of fusion models that are based on ensemble learning strategies to synergize diverse model outputs into one, and a propagation and aggregation scheme that orchestrates the information flow within the knowledge graph. Our Digital Twin design is built upon existing clinical infrastructures across departments, such as the hospital information system (HIS). Within this environment, we define a set of requirements that need to be fulfilled in order to ensure clinical acceptance and broad applicability and, hence, guide the algorithmic design. The design bears five characterizing features. It is modular, informed, predictive, evolving, explainable and interpretable. To enhance clarity, we illustrate specific features using two case studies, prostate cancer diagnosis and glioma treatment, throughout the article. A detailed description of those case studies is given in the supplementary material.

## Fundamental considerations

### Requirements for a digital twin in clinical patient care

The widespread adoption and efficacy of personalized models, including DT models, in clinical practice face several persistent challenges that can be categorized into three types of requirements. The first category includes the specific requirements posed on a Digital Twin in clinical patient care, such as the holistic representation and simulation of the real-world patient journey. Secondly, we can summarize the design requirements necessary for the correct handling of data, dealing with aspects such as data missingness, data inconsistency, multi-modal data types, large datasets^2^. The third category includes requirements related to the clinical acceptance of such a DT system, such as the adaptation to new procedures, robustness, incorporation of existing evidence, continuous learning and interpretability, especially since many machine learning models function as black boxes^7,14^. A more comprehensive overview of the requirements of all three categories for a DT in the clinical context can be found in Supplementary A.

**Fig. 1:**
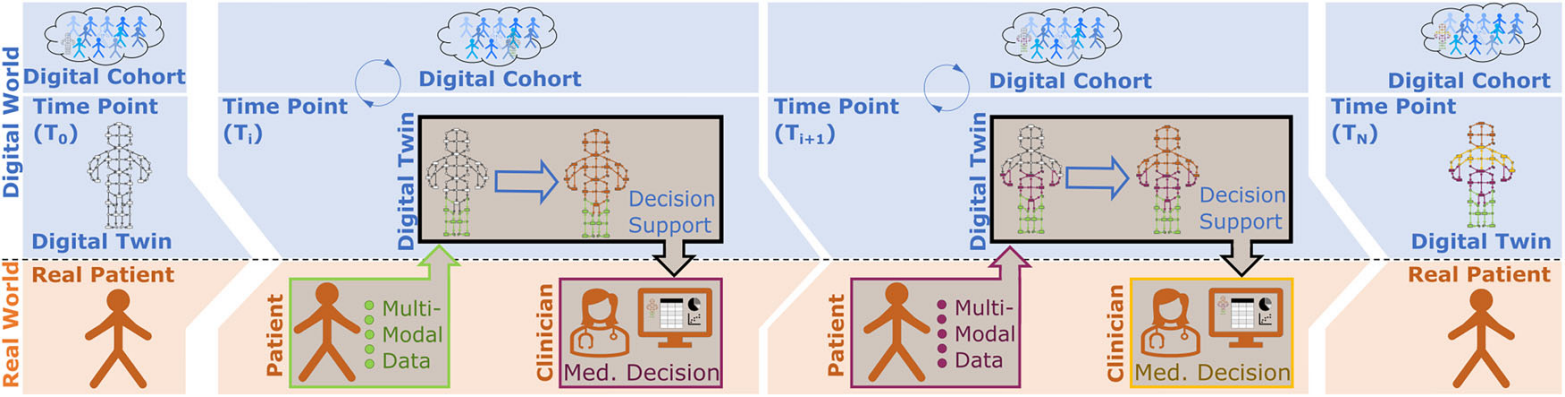
Visualisation of the general design of a DT for clinical patient care, generating an interface between the real world (orange; including the patient and the clinician) and the digital world (blue; including the Digital Cohort - Data Cohort and the DT - patient-specific information and algorithmic structure). Multi-modal data gets transferred from the real patient to the DT, which presents its decision support to the clinician for a medical decision.

Based upon the conceptual requirements from the first category, the characteristics of a DT for clinical patient care are visualized within Fig. 1. Key characteristics and definitions for any DT include the existence of a physical counterpart (one-to-one), the ability to provide a holistic representation, support for bi-directional communication, and the capability for immediate responses and operation upon data entries (real-time data)^15^. In clinical routine, data entries and updates are performed at clinically meaningful intervals (e.g., sub-seconds in surgery, hours in an intensive care unit, or weekly in outpatient settings). At each of these time points *T*_*i*_, a bidirectional communication between the real and the digital world is initiated, by passing multimodal data from the patient to the DT, expanding its patient-specific representation and allowing for predictions or simulations leading to an interpretable Decision Support output.

This information is forwarded to the clinician in the real world to execute a medical decision related to medical interventions that lead to changes in the patient’s state and/or new data acquisition. The collected patient information is included in the Digital Cohort, which is forming the knowledge base for any prediction model within the DT, allowing for continuous learning and extension of knowledge. Additionally, at every time point defined as the update and prediction of the DT, the current representation of the patient state is stored, and newly available data from the Digital Cohort can be used to improve and retrain the DT. The interaction between the Digital Twin and the Digital Cohort can be seen as a second type of bidirectional communication. From a clinical perspective, it is important to emphasize that the DT system will support and supplement current clinical practice instead of replacing rigorously derived clinical knowledge and guidelines.

### Phases of the clinical patient journey

A detailed understanding of the specific contexts within the clinical patient journey in which the DT will operate is essential for developing an algorithmic design that performs reliably across all relevant settings. The explicit structure for each patient journey can differ depending on the disease type, clinical setting, and country. Therefore, an abstract representation of the individual steps within a clinical patient journey and the related decision-making process is needed. We derived the classification of three phases within the clinical patient journey, leading to distinct prediction tasks for the DT.

First of all, there is the *observational phase*, in which complementary attributes of the patient need to be predicted. This can be viewed as a time prediction of the knowledge of the current patient state. The DT can predict the outcome of a measurement based on several models and for which the measurement procedure ideally will not change the current patient state. Two examples for such measurements are provided in the supplementary material C.1. Those are diagnostic imaging procedures and biopsies for cancer staging, each of which provides information that guides treatment decisions.

Secondly, there exists an *active phase* for which the DT needs to predict changes in the patient state related to the performance of a certain intervention. This phase comprises the treatment planning, for which clinicians should be provided with a patient-specific outcome prediction and a risk profile for each potential treatment. The DT’s predictive power and reliability in that phase must be able to be evaluated by clinical endpoints (e.g., survival, recurrence, hospitalization rate). For example, as outlined in the case study of glioblastoma in the supplementary material C.2, the DT can forecast treatment outcomes given a potential chemotherapy or radiotherapy by predicting the overall survival of the patient, effectively simulating what-if scenarios.

Finally, there is a *monitoring phase*, which entails making predictions regarding changes in the patient’s state without any interventions. This can be, for example, the observation of disease-specific parameters after treatment, indicating the recurrence of a disease. The DT continuously tracks, for example, follow-up biomarkers, imaging data, and patient-reported outcomes. Real-time updates of the DT may trigger early detection of tumour recurrence. In the case of a glioma, the treatment follow-up consists of regular MRI scans indicating the recurrence or progression state of the tumour.

## Design of the Digital Twin

### General design considerations

**Fig. 2:**
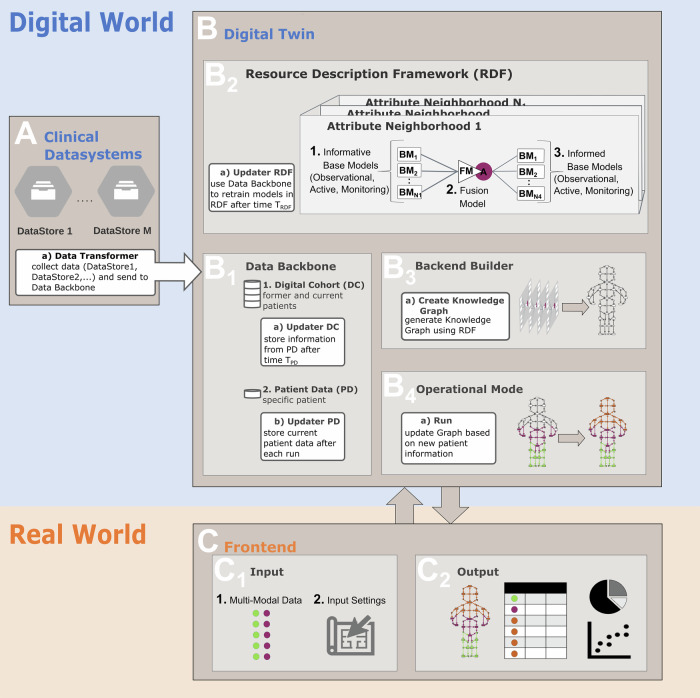
Schematic overview of our proposed software design for patient-centered DTs in Medicine. It consists of 3 different containers, which are labelled **A**–**C**. Those represent the different interacting building blocks, where **A** is the existing clinical data systems (hospital information system) feeding into **B**, the DT itself, explicitly the *data backbone* (B_1_). The *Resource Description Framework* (B_2_) stores all available information about models and their links with attributes, which the *back-end builder* (B_3_) uses to construct a knowledge graph upon which the *operational mode* (B_4_) is going to perform predictions. The user interacts over **C** the *frontend*, where block C_1_ represents the inputs and C_2_ the corresponding outputs from executing the function *run* (within B_4_). More detailed information is given in the text.

Figure 2 schematically depicts our unspecialized DT design for clinical patient care, operating as an overhead to several pre-existing specialized models in a given clinical setting. *Clinical datasystems* (A) include patient data that is collected across several departments and typically accessed via a hospital information system (HIS). To make the patient data accessible to machine learning algorithms, a *data transformer* (A.a) needs to bring the data in a consistent format, as this is commonly collected within and distributed across diverse data formats. The *data transformer* stores the structured patient data in the DT’s building block called *data backbone* (B_1_).

The overall Digital Twin design (B) consists of four components: the *data backbone* (B_1_), the *resource description framework* (B_2_), the *backend builder* (B_3_) and the *operational mode* (B_4_). The *frontend* (C) is presenting the input to the Digital Twin, consisting of the *multi-modal data input* (C_1_.1) and *user settings* (C_1_.2), as well as the DT’s *output* (C_2_), visualising the final state of the knowledge graph-based patient representation. The data modalities can include laboratory tests, vital signs, medications/procedures, images, waveforms, free text, patient-reported data, devices/wearables, genomics, etc.

Specific examples of this design are provided in Supplementary C, for the case of a prostate cancer biopsy (C.1) and glioma treatment (C.2).

### Data backbone

The first building block of the Digital Twin in Fig. 2 is the *data backbone* (B_1_), which consists of the *Digital Cohort* (DC) (B_1_.1), an internal storage of the data of previous and current DTs, and the *patient data* (B_1_.2), an internal storage of all measured as well as predicted states of the current patient of interest. Those two are connected via an *updater* (B_1_.a), which at time intervals T_*DC*_ passes data from the patient of interest to the DC. The *data backbone* is the foundation on which models within the *Resource Description Framework* (RDF) (B_2_) are going to be trained via the *updater RDF* (B_2_.a) after some time period T_*RDF*_.

### Resource description framework and backend builder

As visualized in Fig. 2, the *Resource Description Framework* RDF (B_2_) stores all available information to construct the knowledge graph-based patient representation. A knowledge graph is able to integrate various bits of knowledge (nodes) and their complex interrelations (edges). Two types of nodes exist within our knowledge graph design. The first node type are attributes representing patient data (e.g., biomarker, screening image, genomic mutation, clinical decisions) with their corresponding fusion model. The second node type is base models capable of predicting one set of attributes from another, including computer-interpretable-guidelines (CIG), mechanistic models and AI-driven models. The included base models are mainly support systems, able to solve or predict one highly specific task. In the prostate cancer case study (C.1), such models predict biopsy outcomes, such as high-grade cancer, based on distinct diagnostic features. For glioma treatment response estimation (C.2), base models perform a survival prediction. The directed edges incident at one attribute can be categorised into two types: “informed by” and “informs”. The RDF can be organized into *attribute neighbourhoods* listing all base models that inform (B_2_.1) or are informed (B_2_.3) by an attribute. The *attribute neighbourhoods* have a fully modular structure and form the building blocks used by the *backend builder* (B_3_) to create a bipartite, patient-specific *knowledge graph* (B_3_.a) [ → Modular Digital Twin].

Importantly, our approach is entirely agnostic towards the inner workings of any integrated model, allowing for versatile sources of information, from powerful machine learning models or simulations over computer-interpretable guidelines (CIG) to even human experts. By including literature and practice-based evidence in the form of base models, we are using Informed Machine Learning within our DT design [ → Informed Digital Twin].

Even if there is no difference in how the models are integrated into the knowledge graph, we can make a conceptual distinction between three prediction tasks in the different clinical phases, respectively related to *observational models*, *active models* and *monitoring models*, which will be further discussed in Supplementary B.1 [ → Predictive Digital Twin].

**Fig. 3:**
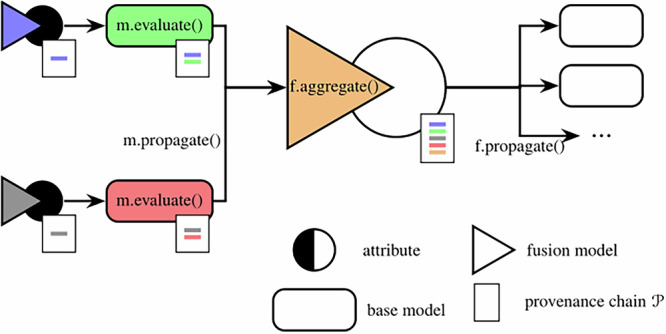
Local structure of the knowledge graph. Outputs from two different models (green and red) are passed on to the fusion model of an internal attribute (circle). Which then gets forwarded to downstream models. The signature propagation from upstream to downstream models is illustrated by coloured signatures on the provenance chain. Labels indicate subroutines from the network algorithm described in Fig. 4.

As evident from the structure of the *attribute neighbourhoods*, several base models can predict one attribute(B_2_.1), leading to redundancy. To address this, we pair each attribute with a unique model, called the fusion model (B_2_.2) according to ensemble learning terminology. It is tasked with aggregating different statements into one consensus value. The simplest case of two proposals being aggregated into one is illustrated in Fig. 3 and forms the fundamental building block of the network. The final attribute prediction can then itself inform other base models (B_2_.3).

The fusion model (B_2_.2) is the central mechanism that extends the Digital Twin’s capabilities beyond those of the underlying base models. Data fusion is well established as a means to improve predictive performance, even when implemented through relatively simple techniques such as weighted averaging of inputs (aggregation mode). Beyond enhancing accuracy, a carefully designed fusion model increases robustness: it can mitigate the impact of implausible or inconsistent inputs by applying robust estimators, predefined plausibility ranges, and clinician-specific weighting that reflects trust in individual data sources. Most simple non-parametric approaches for fusion models, like, e.g., weighted averages for continuous parameters or majority votes for discrete decisions, are genuinely resilient to missing inputs. They can generate outputs from any available data while progressively refining predictions as additional inputs become available. Such incremental integration further enables cross-checking for internal consistency. Failure modes, such as irreconcilable conflicts in binary decisions, become progressively unlikely as the complexity and the number of input decision grows. In the remaining undecidable situations, the fusion model needs to be able to pass such conflicting outputs back to the doctor or at least refuse to propagate the results.

In an exploratory study^16^, we investigated different weighting schemes for the fusion model. The base models were given either by different data modalities, pre-existing models, or different diagnostic procedures. The fusion methods included accuracy weighting, entropy weighting, linear regression, logistic regression and neural network models. For the use cases of heart disease detection and glioma diagnosis, there was no significant performance difference between the different weighting methods^16^.

Further, we illustrate the behaviour of the fusion model using two clinical case studies that represent different fusion tasks, as shown in Supplementary C. These examples range from simple overwriting modes—based on clinicians’ primacy votes—to more complex and comprehensive aggregation across different timescales of therapy options.

### Operational Mode

Attributes determined by real-world diagnostics are used as starting points, activating all available pathways to reveal the most plausible completion of all relevant attributes of the knowledge graph. This update and execution is performed via the *run*-function of *operational mode*’s (B_4_) as shown in Fig. 2). The new input is propagated through the knowledge graph until a maximally holistic attribute estimation is reached. The prostate cancer diagnosis case study in supplementary C.1 illustrates such an update, using an MRI examination as an example. Before the MRI, only one base model could predict high-grade cancer. Afterwards, automatic evaluation of the MRI allows for three base models to predict high-grade cancer, resulting in a much more profound prediction. This state of the current best knowledge of the patient of interest is stored within the *patient data* by the *updater PD* (B_1_.b). This feedback can also be used during the RDF update to adapt or refine the models, constantly improving the DT. Additionally, new *base models* can be added to the RDF, and evidence-based models like CIG can be updated, leading to a constant improvement of the DT [ → Evolving Digital Twin]. The computational demands will linearly grow with the number of base models and the frequency of their reevaluation, i.e., the frequency of new external inputs. The computational overhead of the fusion is small.

**Fig. 4:**
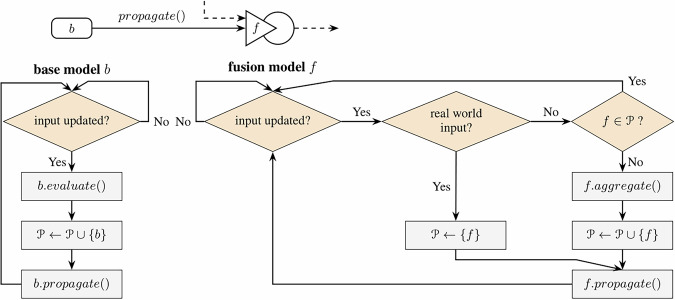
Flowchart illustration of the main network propagation and aggregation scheme in the operational mode. Behaviour differs between base models (left) and fusion models (right). The fusion models only propagate if their respective attribute is set externally or the provenance chain ${\mathcal{P}}$ does not already contain its own signature. Loops can run locally and independently of each other.

The propagation algorithm’s design, as described in Fig. 4, is localized and asynchronous, meaning individual modules can perform their tasks independently. Base and fusion models constantly listen for updates on their inputs received from other models via appropriate transmission protocols. The base models will evaluate and propagate the results if an update is detected and all requirements for model evaluation are met, i.e., all input attributes are available. The propagated outputs are then registered as updated inputs by all downstream models, potentially triggering their evaluation next. This process continues until all reachable branches of the network have been activated and no more changing attributes occur. In terms of computational demands, the asynchronous implementation also means that lighter models are not slowed done by more resource-intensive simulations. Similarly, parts of the network with incomplete inputs do not prevent the evaluation of parts with complete ones.

To keep track of the origin of information, each model adds a unique signature to a provenance chain ${\mathscr{P}}$ that is passed along with the propagated attribute values. In effect, this list contains all attributes and models that have influenced the current value of an attribute. This serves two purposes. Firstly, it is important to maintain a degree of interpretability. Secondly, it addresses feedback loops, which describe an output that is returned to the input and are one of the key features in any complex network. These occur naturally when complementary sets of attributes are connected reciprocally via different models. Loops are problematic, as minor inconsistencies can amplify, rendering the outputs useless. The provenance chain offers a safeguard against this kind of failure.

Based on this provenance chain ${\mathscr{P}}$, the fusion models’ acceptance and propagation of new information are conditioned on a series of checks. Firstly, as shown in the right flow diagram in Fig. 4, if the attribute is externally set, the fusion model will obviously not accept any proposals from upstream Models as it already knows the true value (overwrite mode). It will forward this value with only its signature, marking the external attribute as an origin of information. Further, if a fusion model registers its signature on the provenance chain passed along one of its inputs, it will refuse to acknowledge the update, stopping feedback loops and ensuring the algorithm terminates.

Finally, the *output* (C_2_) of the Run-function is visualized in a user-oriented, intuitive way, enabling insights into predicted attribute values, algorithmic prediction, and performance measures, as well as model-agnostic interpretation methods to allow medical explainability by clinicians [ → Explainable Digital Twin]. Additionally, the graph structure itself is intrinsically interpretable as a type of technical interpretability [ → Interpretable Digital Twin], as the propagated signatures can be used to backtrack the influences of different attributes, as shown in the Supplementary B and by portraying the graph for both clinical cases in Supplementary C.1 & C.2.

## Conclusion

### Clinical translation

In order to translate this abstract concept into a clinical application, several aspects have to be considered. First, validation is essential. Validation can begin with retrospective data, where the predictions of the DT are compared with ground-truth clinical outcomes. Prospective trials or real-world prospective observational studies can then measure whether the DT’s recommendations improve decision-making efficiency, reduce errors, or translate into better patient outcomes (e.g., survival, quality of life). Metrics such as ROC AUC, F1 scores, calibration curves or decision curve analysis can be used.

Second, as medical decision support software, DTs must follow the guidelines of bodies such as the US FDA, the European Medicines Agency, or similar. These guidelines often require transparency regarding model updates, risk analysis and performance metrics. Implementing a robust control system for each model and systematically reporting to regulators will be critical for compliance and transparency. Full compliance with regulatory frameworks, such as GDPR, HIPAA, or Medical Device Regulation, inevitably puts restrictions on specific parts of our design. For future implementations of our design, these regulations have to be properly taken into consideration and may be further refined in the future with the increasing availability of Digital Twins

Third, operability in existing clinical infrastructures is essential. Our approach addresses this aspect by constructing a clinic-specific knowledge graph representation of the patient in accordance with clinical workflows, existing information streams and regulatory aspects.

Fourth, clinical adoption depends on clinicians’ trust and understanding of the DT results. Therefore, we strongly recommend an interactive dashboard for the visualization of the DT design’s intrinsic interpretability, as well as model-agnostic explanation methods (as presented in Supplemental Fig.1). On the liability side, the role of the DT is supportive, not prescriptive: the ultimate responsibility remains with the clinician, and transparency features (e.g., explanations and confidence intervals) help clinicians assess when to rely on the DT versus their own expertise.

### Compliance with requirements

Each feature of the presented Digital Twin design enables compliance with distinct requirements that are posed on such a decision support system in the clinical context. A more detailed argumentation of how the Digital Twin design complies with the requirements mentioned above can be found in the Supplementary B.

A Predictive Digital Twin (through the inclusion of predictive base models) enables decision support along the whole patient journey (within all clinical phases: observation, active, and monitoring). A first type of bidirectional communication between the real and digital world, as well as the immediate response and operation upon data entries (real-time data), is facilitated.

A Modular Digital Twin will allow the implementation of several properties, such as handling multimodal input data, big data, data use optimization, information loss and missing values. Additionally, it facilitates the adaptation to new procedures, as well as reliable and robust predictions.

An Evolving Digital Twin leads to continuous learning (evolution) through a second type of bidirectional communication between Digital Twin instances of individual patients and the Digital Cohort. This simplifies the update of the decision support system and the scalability to new settings.

An Informed Digital Twin that is evidence-adaptive can be generated by including computer-interpretable clinical guidelines (as a type of medical evidence) as base models. The inclusion of Informed Machine Learning using clinical guidelines for final hypothesis validation leads to a third type of bidirectional communication between experts and the Digital Twin.

An Interpretable and Explainable Digital Twin will enhance clinical acceptance by the interpretable algorithmic design of the bipartite knowledge graph, explainable visualization methods, and an interactive output design.

### Limitations

Despite the strengths of the DT design already presented, it has design-specific limitations, like the modularity of the Digital Twin. It can be understood as a trade-off between increasing the relative amount of training data (input feature) and losing information between features by splitting them up. Furthermore, our design relies on the availability of data and specialized base models.

Generally, for any DT, data of higher quantity and breadth needs to be collected to be able to use the DT as a holistic decision support system. A general potential risk for the introduction of biases comes from supervised machine learning, focusing on the identification of structures frequently represented within the data. In the ideal case, hospital information systems from several clinics can be linked together, forming a stronger database to further improve personalized care. In this way, Digital Twins of clinical patients can even be enabled to cover rare clinical cases.

Overall, broad service accessibility of the DT systems needs to be ensured, as it could otherwise be a driver for inequality in health-service provision^17^ and widen a socio-economic gap^18^. Therefore, further improvement, scaling, critical analysis, and risk assessments of any DT designs are crucial.

### Future opportunities

Our scalable approach allows the application to several settings and, therefore, can be applied to a broad variety of medical cases, as exemplified for two cases in Supplementary C ("diagnosis of prostate cancer” and “survival prediction in glioblastoma”). Another example is cardiology, where many mechanistic, evidence-based models are available. Our DT successfully enables the combination of mechanistic with AI-based models. In oncology, e.g., for prostate cancer, multi-modal data is becoming increasingly relevant^19^, and the heterogeneity of the disease leads to a variety of treatment options and patient journeys^20^, which can all be represented in our graph structure.

Beyond cardiology and oncology, DTs have broad potential to improve clinical decision support in many clinical fields, e.g., pulmonology (for managing chronic respiratory diseases like asthma by predicting exacerbations), endocrinology (for continuous monitoring and prediction of blood glucose trends to improve treatment strategies in diabetes), critical care (for real-time monitoring and early-warning systems in intensive care units), surgical planning (for creating patient-specific models to optimize preoperative planning and predict surgical outcomes) as well as infectious diseases (for modelling the spread of infections, which could aid in personalized treatment during outbreaks).

The predictive nature of the DT over several steps in the patient journey can also improve early diagnosis or prevention of diseases^5^. Due to the nature of limited clinical data before a disease, the DT will need large-scale routine medical data with socio-economic and behavioural data. In addition, digital devices, such as wearables that track physiological parameters, can enable a new dimension of predictive modelling. First applications might be common chronic diseases like diabetes or hypertension.

Therefore, given an appropriate environment with the availability of data and base models, our DT can support clinicians and doctors in the prevention, diagnosis, staging, treatment and follow-up of the patient. To improve the acceptability and also to simplify the validation, starting with small building blocks is favourable. This can be a single decision support tool based upon multiple base models or a tool using the combination of two base models through an intermediate attribute. Due to its adaptability, an extension can easily be added later on. The tasks the digital twin can handle will grow with time. At the same time, the interface will stay similar, making adoption easier. We have developed and analyzed a first building block, i.e., for the fusion of CIGs with ML models for glioma classification^16^ and in the case of prostate cancer for diagnosis in order to minimize biopsies.

## Supplementary information

Supplementary Information

## Data Availability

No datasets were generated or analysed during the current study.
