# Supplementary material for: Design for a digital twin in clinical patient care
Source: Npj Health Syst. 2026 Feb 2;3:11. doi: 10.1038/s44401-025-00060-1 (PMC13354220; doi:10.1038/s44401-025-00060-1)
Supplement: Supplementary file 1 — Supplementary Information [file 44401_2025_60_MOESM1_ESM.pdf]

# Supplement - Design for a Digital Twin in Clinical Patient Care

Anna-Katharina Nitschke[1,*], Carlos Brandl[1,*], Fabian Egersdörfer[1], Magdalena Görtz[2,3], Markus Hohenfellner[3], and Matthias Weidemüller[1,†]

[1]Physikalisches Institut , Universität Heidelberg
[2]Junior Clinical Cooperation Unit 'Multiparametric Methods for Early Detection of Prostate Cancer', German Cancer Research Center (DKFZ), Heidelberg, Germany.
[3]Department of Urology, Heidelberg University Hospital, Heidelberg, Germany.
[*]These authors contributed equally to this work.
[†]corresponding author, Email: weidemueller@uni-heidelberg.de

December 8, 2025

## A Requirement for Digital Twin in Clinical Patient Care

As presented in Section 2.1 the requirements of a DT in the context of clinical care can be divided into three categories: Requirements concerning the DT itself, requirements related to data handling, and requirements associated with clinical acceptance. In the following, we identified the relevant requirements that are fulfilled by our design's five main features (Predictive, Modular, Evolving, Informed, Interpretable and Explainable) as introduced in sections 3.3 and 3.4 of the main text. The requirements are based on the discussion presented in Schwartz et al. [1] and Kelly et al. [2].

**DT requirements**

- R1 Holistic representation of the patient journey
  - R1.1 Operate in observational phase
  - R1.2 Operate in active phase
  - R1.3 Operate in monitoring phase
  - R1.4 Enable the prediction of the time evolution of parameters
- R2 Facilitate a bi-directional communication
- R3 Responds and operates upon data entries (real-time)

**Data handling requirements**

- R4 Multi-modal input data
- R5 Deal with big data
- R6 Optimize data use
- R7 Avoid information loss
- R8 Handle missing values

**Acceptance requirements**

- R9 Ease adaptation to new procedures
- R10 Achieve reliable and robust predictions
- R11 Continuous learning
- R12 Update decision support system
- R13 Ease integration into clinical workflow and scalability to new settings
- R14 Informed by medical evidence
- R15 Minimize human barriers
  - R15.1 Enable interpretability
  - R15.2 Facilitate explainability

## B Features of the Digital Twin Architecture

In the following we specify desired features and discuss how our proposed structure is dealing with the requirements introduced in Part A by advantageously combining several methodologies making up the core features of a DT that is **Predictive**, **Modular**, **Evolving**, **Informed**, **Interpretable and Explainable**.

### B.1 Predictive Digital Twin

> **F1: Predictive**
>
> The inclusion of predictive base models (clinical phase-specific: observational, active, monitoring) enables decision support and time evolution simulation along the whole patient journey. A first type of bidirectional communication between the real and digital world in real time is facilitated.

Our Digital Twin design is the first to explicitly follow the patient journey through different medical decision time

points. The modularized design of the network algorithm facilitates the integration of diverse base models ($B_2.1$ & $B_2.3$), covering a variety of prediction tasks. Similar to the model for a structural DT of an unmanned aerial vehicle presented by Kapteyn et al. [3], distinct time phases can be distinguished. They defined one phase in which complementary information gets observed to better understand and describe the physical asset, called the calibration phase. In our case, this corresponds to the observational phase for which a change in the knowledge about the patient state is performed. Kapteyn et al. defined a second phase, called the operational phase, in which a fixed set of parameters is observed over time. We additionally distinguish the two model prediction tasks for which a change in the patient state can occur. One would be due to the performance of an intervention (like treatments), described as the active phase. Second, a change in the patient state due to parameter evolution (like active surveillance), described as monitoring phase. Hence, equivalently to Kapteyn et al., depending on the current phase different methods can be used to connect the information gained over time and connect the different steps of the patient journey.

**R1.1: Observational Phase -** An increasing number of parameters are observed to better understand and precisely describe the patient's health state. For example, different screening methods can be used in the diagnosis phase. After each information gain, the patient's health state is estimated, and the question is asked whether the patient shows a high probability of the need for treatment and needs to be passed to the next step within the patient journey. The reflection of this procedure in the digital world can be understood as a form of Bayesian inference, as mentioned by Kapteyn et al. [3]: "Using this observed data, we perform a Bayesian update on our prior estimate in order to produce the posterior estimate [...]." At each time point, there is a prior belief about a patient's state parameters, which are adjusted according to new insights, progressively individualizing the DT. In this phase, the models that comprise the DT structure at each time point, which include data-driven models, need to be trained on different parameter spaces and hence are independent from one another.

**R1.2: Active Phase -** During this phase, the patient undergoes one or more interventions. A more or less consistent set of parameters is monitored over time, reflecting the patient's health status and any changes induced by interventions. By directly connecting the DT to clinical monitoring systems, this can happen in real-time. Predicted attributes will either encode recommendations for future interventions or anticipate their outcomes. Examples of such attributes include the likelihood of disease recurrence or the quality-adjusted life years, which balance the quantity and quality of the patient's remaining life [4]. Further instances of active phase modelling are Capelli et al., who explored computational models for treatment planning in congenital heart disease [5], or Smith et al., who have developed a personalized treatment planning model that integrates physical, biological, and clinical factors to optimize IMRT plans for prostate cancer [6]. By adding attributes reflecting not only the patient state but also the state of the clinical workflow, our network-based approach allows for the flexible modelling of a potentially very complex decision logic, where the performance of future interventions is conditioned on the steps taken in the past. This path dependency is often present in clinical workflows. The corresponding models must account for attributes that indicate prior interventions to achieve this. Many existing models, however, provide only general predictions about future developments. In such cases, their outputs can be incorporated into the network like any other attribute, with the implicit understanding that these projections represent future scenarios.

**R1.3: Monitoring Phase -** In the monitoring phase, a change in the patient state without the performance of an intervention is needed. In the clinical context, this is the case for monitoring in-patients, e.g., in intensive care units, as well as for out-patients, e.g., for monitoring the patient's health status after a treatment has been completed. For in-patients, real-time prediction based on sensory data is a typical task [7]. Examples include the prediction of sepsis [8], near-time mortality prediction [9], and prediction of cardiac arrest in emergency departments [10].

For outpatient care, the monitoring phase typically corresponds to the final part of the clinical patient journey after treatment has been successfully completed and the possible recurrence of the disease is of interest. Such outcome prediction can take various forms. Especially in oncology, cox-regression models predict the probability of progression-free survival for a time period [11, 12]. In general, clinical outcome prediction aims to detect deterioration of the patient, such as cardiac arrest, mortality, or intensive care unit (re-)admission [13]

Apart from data-driven prediction models, this also includes mechanistic models to simulate biological processes. For the application considered here, the most appropriate method has been presented by Masison et al. [14] as they have been developing a hub- and-spoke modular design for their simulation-based medical DT. They presented a simulation solver that could alternatively be used instead of the Fusion method and orchestrates the execution of individual submodules in the biological process.

**R1.4: Time Evolution of Parameters -** A model task that can occur within the active and monitoring phase is the prediction of the time evolution of certain clinical parameters, crucial for further patient journey. Models provide a continuous prediction of the temporal evolution of certain attributes and can give predictions at variable times. For these, an extra time attribute can be introduced that the clinician can manually set. The outputs from these models now effectively live in their own time zone that the propagation algorithm is not (and does not need to be) aware of. In principle, a complete copy of the network can be attached to the new time-projected attribute,

which, utilizing the usual propagation rules, would project the entire patient state to the new time zone. A variety of methods is known for clinical time series prediction, ranging from simpler statistical models like Autoregressive models, Linear Dynamical System models, and Gaussian Process models (discussed by Liu and Hauskrecht [15, 16]) to complex biophysical simulations (see Chase et al. [17]). The best-suited method for each setting depends on several factors like the time intervals, the parameter types, the type of noise, and the prediction or detection goals. Kapteyn et al. [3] mathematically described this phase by *sequential Bayesian inference*, on the output prediction of the specific models at one time point that in the end enables key capabilities of the DT.

**R2: Bidirectional Communication -** Our presented algorithmic structure of a DT serves well for all clinical patient journey phases. Hence, a fundamental requirement, the prediction of the patient state over time, is fulfilled. Through the inclusion of clinical patient data into the Digital World and by transferring the DT prediction through the dashboard into the real world, bidirectional communication is facilitated.

**R3: Real Time Data -** An important quality of a patient DT is the ability to enable bidirectional communication in real time. In clinical routine, real-time often implies data updates at clinically meaningful intervals (e.g., subseconds in surgery, hours in an intensive care unit, or weekly in outpatient settings). Therefore, the link to the different clinical data systems must be established. An approach for a DT platform to establish such a connection on a software framework level has been proposed by Petrova-Antonova et al. based on different web services [18]. With our DT design, we do not impact the information flow or data collection from the patient to the data base (hospital information system). The focus of our design is to ensure that the DT is presenting the best possible representation of the patient. Therefore, the operational mode of the DT is triggered as soon as new information about the patient becomes available.

### B.2 Modular Digital Twin

> F2: Modular
>
> A modular decision support system will allow the implementation of several properties, such as handling of heterogeneous input/mixed-type data; big data; information loss and missing values; data use optimization; scalability /adaptation to new procedures; reliable and robust predictions; validation.

As visualized in Figure 2 of the main text, the patient-specific DT is configured by the *backend builder* ($B_3$). The generated bipartite knowledge graph links patient attributes and models. The *attribute neighborhoods* are best described as an ensemble learning approach, which is modular by its nature. Thus, each base model ($B_2$.1 & $B_2$.3), processes a part of the known parameter space. In a second step, a final combination of the module output is given through a fusion model ($B_2$.2) that returns an overall prediction. In the following, the different challenges tackled by the modularity of our ansatz are further discussed.

**R4: Multimodal Input Data -** Prediction tasks on multimodal input data are still active research area in Machine Learning. Medical decisions are made on a variety of data, which are of different data formats. Combining these multimodal data for medical decision support systems is not straightforward, as algorithms usually process only one class of data (text, image, numbers, etc.). By using the modular approach to combine the predictions made on different parts of the data, each base model in the *attribute neighborhood* can process its corresponding data type. Current approaches to deal with mixed or multi-view data are reviewed by Li et al. [19]. They presented a general overview of how data fusion can be included through machine learning techniques categorised into early, intermediate, or late integration methods. In early integration methods, features from different data are concatenated into a single feature vector before fitting an unsupervised or supervised model. As this approach seems straightforward and intuitive, constructing a model that is able to deal with this kind of input vector is not easy, as further feature preprocessing might be needed. The intermediate approach involves data integration in the learning process and into the model design. The late integration method, for which separate models (base models) are first trained on the individual data subsets, involves a combination of those individual outputs to a final response and is comparable to our approach, which uses fusion methods (B.d.3).

**R5: Big Data -** As more and more data is generated, the computational power for processing this data needs to be increased. One solution is to diversify the data analysis by training models only on parameter subsets of the whole data. Therefore, less computational power is needed for training the base models, as each of them only works with a subspace of the total parameters.

**R6: Data Use Optimization -** At the same time, our approach helps optimize data use. By dividing the feature space, the relative training data available per feature is increased. Thus, the approach also works in cases where only a limited number of patients are available, like for rare diseases. Additionally, some patients only undergo specific procedures or have a high ratio of missing data, so only very limited parameters can be added to the subsets accordingly. This can further increase the amount of training data per feature subspace.

**R7: Information Loss -** Often, when building clinical decision support tools, not all available features are used by the developed algorithms. The features are usually selected by having prior knowledge of the clinicians or by using feature selection algorithms. But some of them could contain so far unknown meaningful information, which is

why their inclusion could be advantageous. As the precise representation of the current physical condition of the patient is sought, all available information should be used in the best possible way. Hence, additional base models could be trained upon available but unused parameters and added to the fusion model. Thus, our modular approach reduces the risk of dismissing potentially valuable data.

**R8: Missing Values -** Our DT structure can deal with missing values, missing measurements, or measurements being executed in a different order, as the structure of the algorithm can be adjusted very easily. In the case of a predictive task based on multiple base models, missing values would lead to the corresponding base models not being executed. But due to the redundancy by other base models, it would be just the fusion model ($B_2$.2) that needs to adapt to a different number of inputs. Especially if the used fusion model is linear, this process would be resolved by a simple renormalization of the base model weight (see publication Clinical Decision Support System (CDSS) by Brandl et al.[20]). Usually, for other classification algorithms, a problem specific adjustment needs to be made to deal with this problem. Our approach is, therefore, easily generalizable, model-agnostic, and unspecific.

**R9: Adaption to New Procedures -** The inclusion of new decision support tools and practices is a tedious and time consuming process. The modular approach is making the implementation of a newly developed algorithm easier for CDSS developers, as they can use the existing infrastructure. As the software interface does not change, the adaptation to new procedures is simplified for clinicians. Furthermore, less implementation effort is needed.

**R10: Reliable and Robust Predictions -** An important requirement for a medical support system is a reliable and robust prediction. As ensemble methods take into account several opinions and votes of different classifiers, their variance might decrease through averaging over these stories (as shown by publication Brandl et al. [20]). Instead of constructing the best possible machine learning method, the approach is to use the wisdom of the crowds, in which independent classifiers form one decision together. The improvement by using Mixture of Experts is well described in the Literature [21, 22].

### B.3 Evolving Digital Twin

> F3: Evolving
>
> A second type of bidirectional communication between Digital Twin instances of individual patients and the Digital Cohort will lead to continuous learning (evolution).

**R11: Continuous Learning -** Bidirectional communication between individual patients' DT instances and the DT aggregate covering the full patient cohort can be realised by continuous learning/ online learning techniques. The modules in the *RDF* ($B_2$) would therefore need to be retrained after a specified time period on the updated *Digital Cohort* ($B_1$.1), which itself is updated by new *Patient Data* ($B_1$.2). With each iteration the data foundation for the base models ($B_2$.1&3) and the fusion models ($B_2$.2) improves. This process of iteratively retraining the models is called online learning and is in our design controlled by the Updater RDF ($B_2$.a). The whole process can be automated after data quality is ensured. In this way, the DT can additionally adapt to slowly changing factors in the clinical patient population in a temporal manner, like an aging society, resulting in an overall higher mean age.

**R12: Updating Decision Support Systems -** Other decision support tools usually need to get updated regularly to include new characteristics found or changes in the population. The update is generally associated with repeated implementation of clinical studies, resulting in lots of work and costs. By using online learning, the algorithms used for the DT can adapt over time. This is done by regularly including new patients into the *Digital Cohort* and retraining of the model.

**R13: Scalability to New Settings -** The proposed architecture is a general approach to clinical patient care, independent of an explicit clinical setting and explicit knowledge about the disease processes. The DT architecture is adapted to a specific application by choosing suitable base models and data systems. Therefore, the presented DT design is very flexible and can easily be used for a variety of medical patient journeys as well as different medical fields. Through online learning, one can further advance the algorithm to handle new populations from different countries, ethnicities, or even just different institutions. A recalibration would be generally necessary if the solution were implemented for a broader patient cohort, as otherwise, there might be the problem of sampling bias due to intrinsic and extrinsic demographic heterogeneity of the training data [23].

### B.4 Informed Digital Twin

> F4: Informed
>
> A Digital Twin that is evidence-adaptive can be generated by including computer-interpretable clinical guidelines as base models. The inclusion of Informed Machine Learning using clinical guidelines for final hypothesis validation leads to a third type of bidirectional communication between experts and the Digital Twin.

**R14: Informed by medical evidence -** It has so far seemed as if evidence-based knowledge and knowledge extracted through machine learning from data would face each other, although the combination of both seems promising and is desired [24, 25]. In accordance with the discussion provided by Brandl et al. [20], we aim to generate a DT that includes practice-based evidence, e.g., in the form of

computer-interpretable clinical guidelines (CIG). There, we explained the possibility of using multiple base models to validate the guidelines, which can be controlled by increasing or decreasing the guidelines' weight in the fusion model. In case of different opinions on guidelines and remaining base models, a reasoning as to where these differences come from will be helpful for the clinicians. Therefore, the interpretability and explainability (R17) of the individual models will help clinicians to make an informed decision.

An evidence-adaptive CDSS can be generated by including CIG as a base model ($B_2$.1&3) within the graph structure. Hence, Feature 4 "Informed" of our proposed DT design is based on Feature 2 "Modularity", but will be discussed separately due to its importance for the CDSS. The fusion models can effectively model the degree of trust in the guidelines by increasing or decreasing the guideline's weight, which offers a new possibility to validate different Machine Learning algorithms or evidence-based nomograms, as the fusion model calculates the weights of an algorithm in the ensemble based on its performance. Additionally, the DT could also learn to only operate within the boundaries of the guidelines, as the result of the *Operational Mode* $B_4$ can be filtered to comply with the guidelines.

Internally, the discrepancy between the prediction and the guidelines will then be evaluated post-intervention and could result in new insights into the disease. The adaptation of the guidelines and, therefore, the evolving state of knowledge, is easily possible for the DT. A holistic approach is formulated that tries to make the best out of all available information. Therefore, Informed Machine Learning by using clinical guidelines for final hypothesis validation will lead to a type of *bidirectional communication between researcher/experts and Digital Twin*.

Additionally, one can determine if an algorithm performs better on a small group of patients than the total cohort, which offers a possibility to detect bias in that algorithm.

Furthermore, the validation of the current guidelines is possible as we will explain in the next paragraph.

### B.5 Interpretable and Explainable Digital Twin

**F5: Interpretable and Explainable**

The clinical acceptance of the Digital Twin will be enhanced by interpretability in the algorithmic design due to the knowledge graph structure and explainability based on visualization methods and an interactive output design.

**R15.1: Interpretability -** In the medical context, algorithms' interpretability and explainability are mandatory. Interpretability is a technical property of algorithms [26], thus it has to be considered in the design of the Digital Twin. The base models and fusion models in our knowledge graph can be interpretable. For some examples, we have shown that more complex, non-linear fusion models do not necessarily lead to better predictive power [20]. The causal hierarchy between different parts of the knowledge graph can be extracted from the provenance chain passed along with all predictions. These allow the identification of used features and tracking the influence of the different base models. Subnetworks contributing to an attribute's prediction can be visualized, and the user can be given the option to disable branches that seem to influence the attributes in question toward subjectively implausible results.

Although using a modular framework to gain insights and interpretability seems straightforward, there is not much literature on the topic. This might be due to the fact that a mixture of experts framework does not include the generation of (distinct) feature subspaces. Instead, all base models work on the same full parameter space, and the individual instances (patients) are assigned to a model. For that case, it has been shown that using only interpretable models, like Logistic Regression or (Soft) Decision Trees as Base Models and a Deep Neural Network as Assignment Network/Gating Network achieves similar performance to black-box models [27]. As the models are interpretable by nature, a partially interpretable Algorithm on individual instance and feature level is constructed. However, in that approach, it is not possible to manually ex-/include individual models.

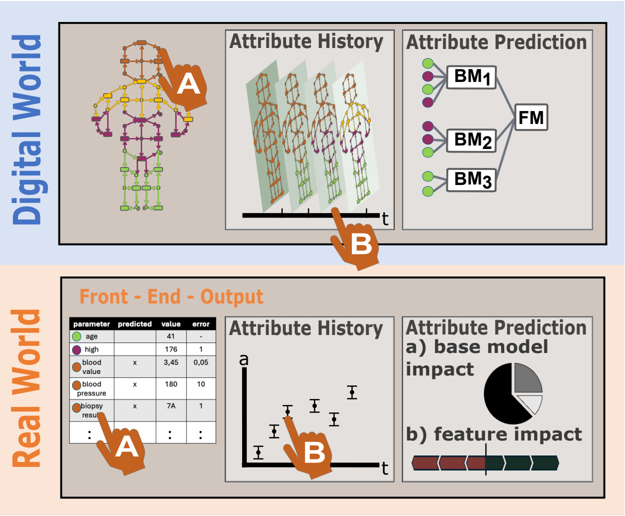


Figure 1: Visualisation of the medical Interpretability by the visualisation of the attribute history and base model impact, as well as feature impact on the attribute prediction. In the digital world, this corresponds to: the selection of a node of the knowledge graph (A), the selection of one execution of the run-function in the Operational Mode (B) and the attribute prediction via an ensemble model.

**R15.2: Explainability -** Beyond interpretability, a user-based property called explainability [26] can be generated by technical tools, helping the user to understand the

model, or can be developed by the user on their own in an interactive manner.

Technical tools will be used in settings in which non-interpretable models have to be used for performance aspects. These are model-agnostic (and model-specific) explainability methods, which are well-summarised in literature [26, 28].

Another way of achieving explainability is by an interactive design, allowing the user, i.e., the medical doctor, to understand the model and develop their own intuition. This can be achieved by a well-designed dashboard. The dashboard output presented in Figure 1 as "Front-End-Output" shows, for example, how an attribute's prediction is impacted by the base models and the attributes through, e.g., SHAP plots [29]. The proposed approach, using a modular structure, introduces the ability to easily interact with the algorithm's structure, allowing the physician to develop an intuition about its decision-making process. This is represented in Figure 1, which shows an example dashboard for the clinician in the real world. The clinician is able to see which values are predicted or measured, the value and its error (A). If several values have been measured or calculated, the history of the attribute value can be visualized (B). For each time-point, the base model impact (a) and feature impact (b) on the attribute prediction can be visualized. The clinician can also directly test hypothetical scenarios (e.g., "What if the patient's age is 10 years older?"). For clinical translation and acceptance, user testing with clinicians to refine the front-end, interpretability features, and iterative improvements is elementary. Figure 1 additionally visualizes what this type of interaction represents on the algorithmic level: the selection of a node of the knowledge graph (A), the selection of a completed graph after the performance of the run-function in the *operational mode* (B) and the attribute prediction via an ensemble model.

## C Clinical Examples

Our first example represents the observational phase of the clinical patient journey, in which information is collected from the patient via diagnostic procedures and the patient's health state can be viewed as more or less stable. The example is showcasing the process of an oncological diagnosis before biopsy, in the case of prostate cancer. As a second example, we outline a case for survival prediction of a glioblastoma patient to exemplify the application in the later stages of the clinical patient journey, representing what we have defined as the monitoring phase.

### C.1 Diagnosis of Prostate Cancer

In our example scenario, we assume a "Patient John Smith" is receiving standard screening procedures in a clinical setting in urology, which include the performance of an anamnesis, laboratory tests of blood markers (such as PSA) and a digital rectal examination (DRE). As a next step, imaging using MRI is performed to identify potential lesions for an MRI-guided biopsy [30]. From the MRI screening, information including an overall score of the malignancy (PI-RADS), prostate volume measurement and additional radiomics features are collected. Based on the results of all screening procedures and predefined clinical guidelines, the responsible clinician needs to assess the need for the performance of such a biopsy. The aim of a biopsy is to better assess the stage and malignancy of a potential prostate tumor. Therefore, a grading called the Gleason score (GS) is performed by the pathologists from the tissue samples that are collected via the biopsy. As there are several potential complications and a biopsy is an invasive procedure, it is desirable to avoid unnecessary biopsies. Therefore, there are many risk prediction models available that aim to estimate the risk of a high GS through the analysis of available patient information [31].

In Figure 2, we have depicted this information flow as follows. Information about the patient is generally collected and governed within the *clinical datasystems* (A) (Hospital Information System - HIS) in an unstructured and distributed way. For example, documentations of anamnesis might be in Word or PDF format. The *data transformer* extracts and structures the patient information from each source and transfers it into the Digital Twin's *data backbone* ($B_1$). An implementation of the *data transformer* has to support a variety of widespread data protocols, as full interoperability in the healthcare context has not yet been reached [32]. Features extracted from the anamnesis could be the family history regarding prostate cancer and other conditions, as well as the performance of prior biopsies. For our example, the data of "Patient John Smith" (including PSA value, DRE results and age) is stored in the *data backbone* ($B_1$), together with the newly acquired MRI information that is additionally represented in the *front-end* as *input* ($C_1$).

For the DT to mimic the information flow of the clinical patient journey, the *attribute neighborhoods* within the *Resource Description Framework* ($B_2$) have to be defined. Figure 2 shows two examples for *attribute neighborhoods* that store the information on what informative base models enter the attribute-specific fusion model, as well as what base models are informed by the attribute. The first exemplified attribute is the PI-RADS score, for which two informative base models are entering the fusion model: the radiologist, represented as "external input" model, as well as the output of an AI-driven "Image Analysis" model - trained to predict the PI-RADS score based on the original MRI images. In such cases, where external and model-based inputs are available, the fusion is defined to select the external input ("overwrite mode").

Two base models are informed by the PI-RADS attribute, namely a radiomics model and a risk calculator, which will be explained in more detail later. The second exemplified attribute is the "high Gleason score", which corresponds to the next step in the clinical patient journey. Therefore, models that are defined as informed base models for the *attribute neighborhood* of PIRADS are now part of the

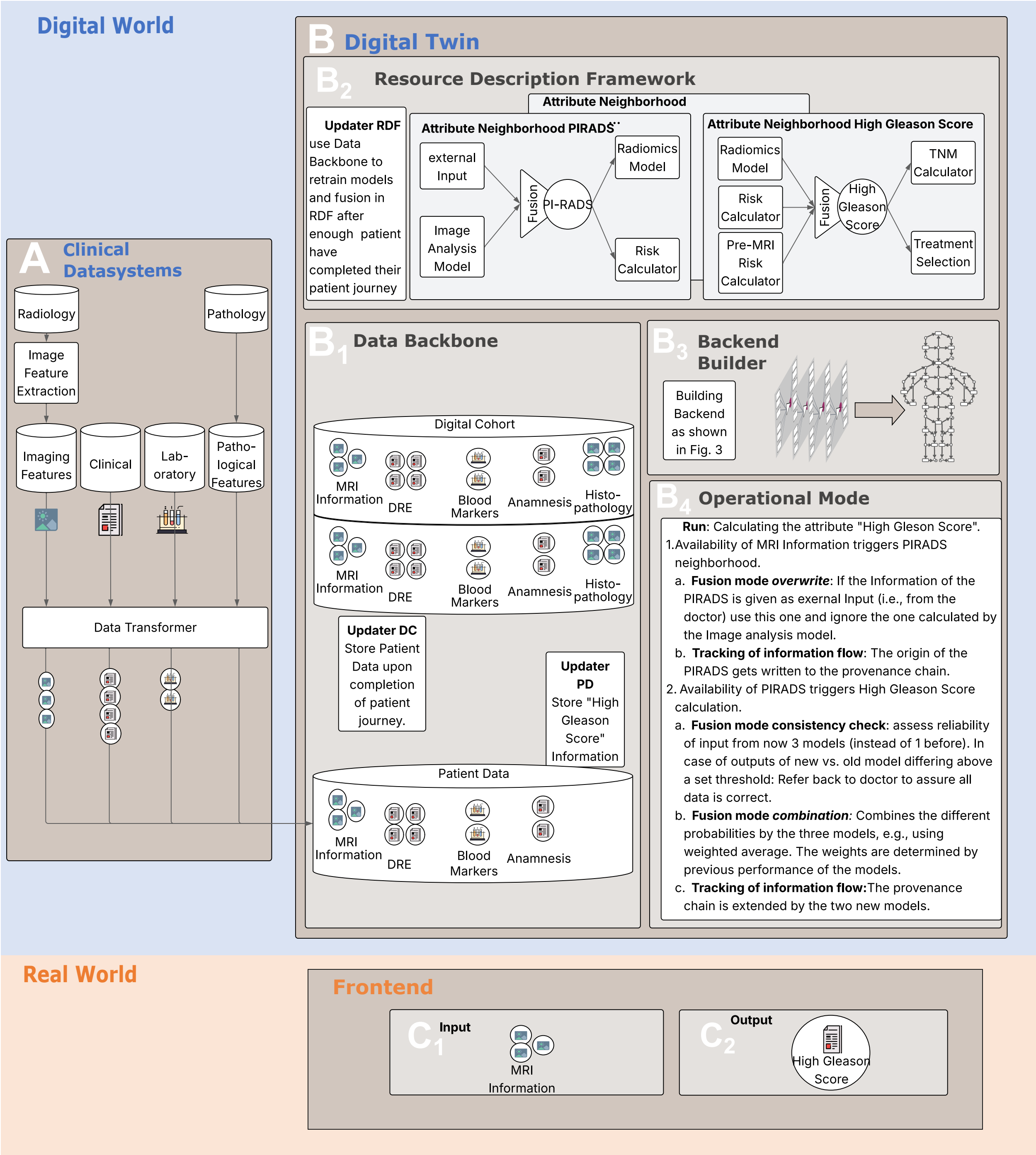


Figure 2: Schematic overview of our proposed software design for patient-centred DTs in prostate cancer diagnosis. The figure is similar to Fig. 2 of the main text, showing the Clinical Datasystems (A), the DT itself (B), and a Front-end (C). The DT consists of the *data backbone* ($B_1$), the *Resource Description Framework* ($B_2$), which stores all available information about models and their links with attributes, which the *back-end builder* ($B_3$) uses to construct a knowledge graph upon which the *operational mode* ($B_4$) is performing predictions. Structured data is visualized as small circles, with the icon depicting the original data source of the clinic. The full knowledge graph for our example is shown in Figure 3

.

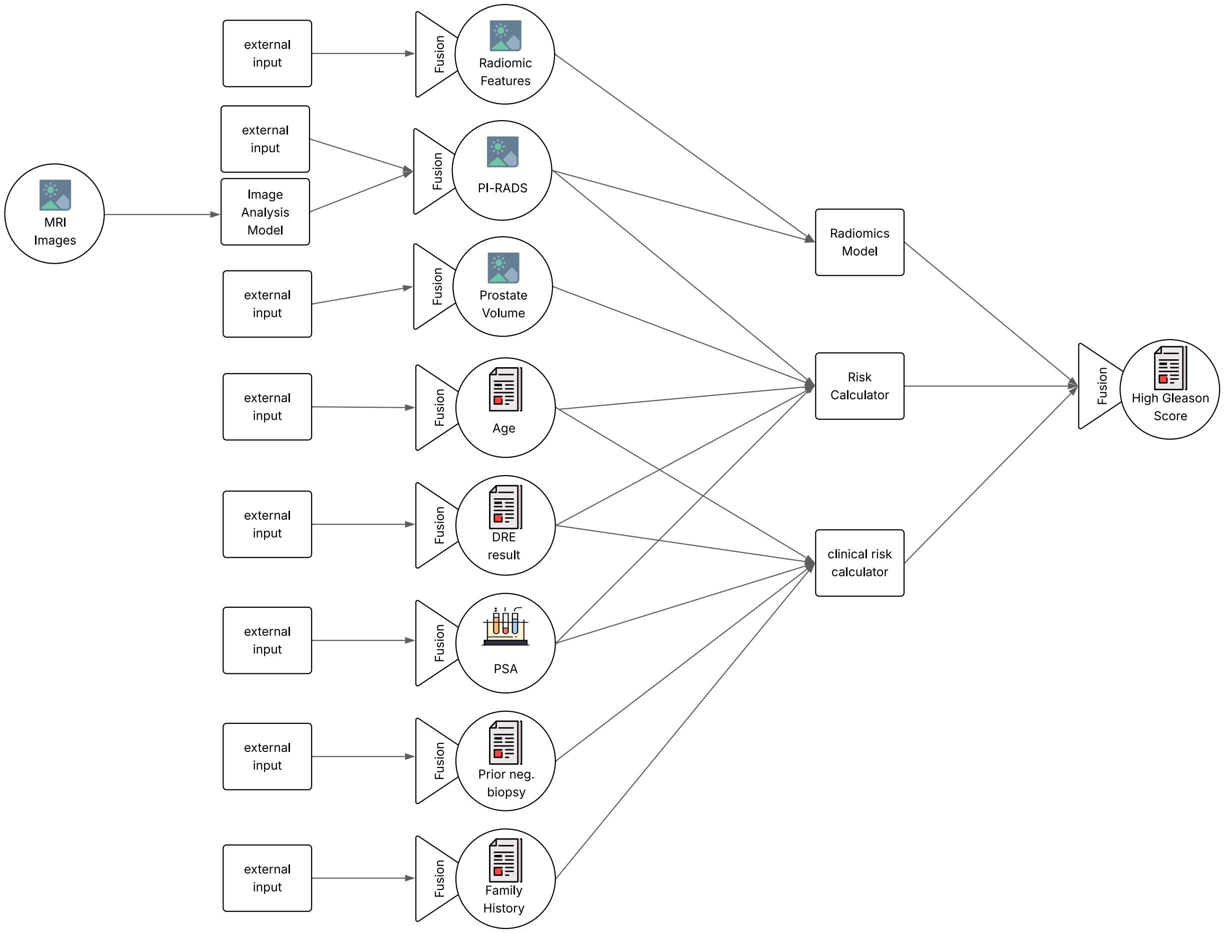


Figure 3: Detailed representation of the knowledge graph for "Patient John Smith" in Figure 2 before a prostate biopsy as constructed by the *Backend Builder* ($B_3$). On the left, the input features are shown as circles, and the output attribute "High Gleason Score" is shown on the right. The models are depicted as squares. "external Input" models

informative base models alongside a new machine learning based model. It is now clear how to combine both *attribute neighborhoods* in the *backend builder* ($B_3$). The PI-RADS will inform the radiomics model and the risk calculator will predict the high Gleason score attribute.

Figure 3 visualizes the knowledge graph that is constructed upon the two introduced *attribute neighborhoods* by the *backend builder*. To simplify the example, we restrict the DT to three specific models, enough to show the main features of our operational mode. All of the models predict the probability of clinically significant prostate cancer. The first model is a model based purely on radiomics data, e.g., Jing et al. [33]. The second model is a mixed model, using clinical data and MRI information in the form of the PI-RAD score [34]. The last model is only using clinical and anamnesis data, i.e., age, psa value, DRE result, family history and whether a previous biopsy was negative. Such a model was developed, for example, by Ankerst et al. [35].

Before MRI information is available, only the clinical risk calculator has all the necessary data available. Thus, only this model propagates information to the high Gleason score attribute. The corresponding fusion receives information of this one model and does nothing more than adding the model's signature to the provenance chain (introduced in Figure 3 of the main) $\mathcal{P}$ of the high Gleason score attribute.

As the PI-RADS becomes available, the fusion model of the PI-RADS attribute notes the origin of the attribute PI-RADS in its corresponding provenance $\mathcal{P}$. As now a PI-RADS is available, the base models radiomics model and risk calculator check if all their inputs are available. In this example, this condition is fulfilled. Therefore, the models evaluate their inputs and provide an output (high Gleason score attribute). Additionally, according to the scheme in Fig. 4 of the main text, the models take the provenance chains of their inputs, combine them, add themselves. On the left side of Figure 4, this is depicted as $\mathcal{P} \leftarrow \{b\}$. The provenance chain gets passed together with the outputs to the fusion model. The corresponding fusion model detects updates of its inputs and as the input is not yet on the provenance chain of the high Gleason score attribute,

the fusion model recalculates this attribute. This is done by a weighted average mechanism, using the previous performance of the models on similar patients as weights. Then it combines the provenance chains of all new inputs and adds them to the existing provenance chain of the attribute high Gleason score. On the right side of Figure 4 of the main text, this is included as $\mathcal{P} \leftarrow \{f\}$. This cycle then continues downstream, but for our example, we stop here at the first iteration. A fully implemented knowledge graph with this propagation and aggregation scheme would stop, either if no base model can be evaluated, because inputs are missing at this stage, or if a loop is detected, i.e. a model finding itself on the provenance chain.

The calculated probability of the high Gleason score attribute will be presented to the clinician. This is the decision support. We expect the clinician to have the option to access all other attributes and their history, independent of whether they were calculated or measured values. Additionally, the provenance chain of the attribute enables tracking of the information flow, which can be visualized in a graph structure similar to Figure 3.

The *updater PD* (in $B_1$) stores the newly calculated information in the patient data. Once the patient has completed the patient journey (or just the current phase of the patient journey), the *updater DC* stores the full patient information in the digital cohort. This time is different for every patient. The shortest period in our scenario would be the evaluation point of the biopsy, i.e., after a few days. At this time, the real value for the attribute is determined. After several patients have been added to the digital cohort, the *updater RDF* updates the models in the RDF. The time interval depends on many factors, mainly hospital size and number of patients, but we can assume it to be in the order of once per year or less. Thus, for our "Patient John Smith", the doctor can now evaluate the predicted probability for a high Gleason score and, therefore, if he needs a biopsy or if it would be reasonable to further surveil PSA values.

### C.2 Survival Prediction in Glioblastoma

In this scenario, we are assuming a patient with glioblastoma multiforme (GBM), which is the most aggressive type of primary brain tumor. To support personalized treatment and avoid ineffective treatment, an accurate prediction of prognosis and survival is crucial [36].

Traditionally, survival prognosis is based on a limited number of molecular features, e.g., MGMT methylation (methylation of the MGMT gene promoter reduces the production of the MGMT enzyme, which is a DNA repair protein that stronly reduce the patient responds to chemotherapy drugs like temozolomide), or clinical features, e.g., age and Karnofsky performance status (KPS describes the patient's physical state and abilities) [37]. Additionally, in recent years, a variety of machine learning based models for survival prediction have emerged, based on combinations of MR Images, radiomic, molecular, genomic, and clinical features [36].

We now exemplify the DT for survival prediction, after the initial treatment for "Patient Sarah Jane", which was a surgical resection of the tumor. For the clinicians, the next decision is if therapy will be continued and, if so, if it will be chemotherapy or radiotherapy [37]. Therefore, models that estimate the survival based on the potential therapy types are needed. The information that has been collected to this point includes MRI scans, clinical data, and surgical outcomes, e.g., resection status. The evaluation metric would be a prolonged survival.

We explain the flow of data starting at the *clinical datasystems* (A) in Figure 4. Similar to our previous example, we assume the *data transformer* to transfer the information retrieved from the *clinical datasystems* in a structured form in the *data backbone* ($B_1$) of the Digital Twin (B). The data consists of imaging information, anamnesis, genetic features, molecular features, and potential previous treatments. After initial surgical resection of the tumor, the surgical margins are known ("resection status"), and the imaging procedure is repeated. Based on this information, the Digital Twin now calculates the survival estimate of our patient, without any further therapy. The doctor can input ($C_1$.2) a treatment option to simulate "what-if" scenarios for treatment. In the *RDF* ($B_2$), the *attribute neighborhood* of the radiotherapy is shown on the left side. It has only the external input, which can be set by the doctor, and informs two models. On the right side of the RDF ($B_2$), the *attribute neighborhood* of the survival attribute is shown, with several base models informing this attribute. To emphasize the scalability of our approach, we have chosen a total of 6 base models, all with slightly different input and output features. A lot more models for predicting survival are available as reviewed by Poursaeed et al. [36].

The first model we chose is from Chen et al. and estimates the survival in days based on radiomic features and clinical features [38]. The second model was developed by Fathi Kazerooni et al. and categorizes patients into high risk (survival < 6 months), medium risk (survival 6-18 months), and low risk (survival > 18 months) based on radiomic features, molecular features, and clinical features [39]. The third model, by Yang et al., predicts survival categories of 1 year, 2 years, or 3 years based on age, KPS, MGMT methylation status, and chemotherapy status [40]. The fourth model was developed by Tang et al. and predicts the survival in days, molecular features, in particular MGMT methylation, and genomic mutations. The prediction is based on a combination of MRI images, radiomic features, and clinical features [41]. The fifth model is by Senders et al. and uses information on therapy, age, and resection status as inputs to predict a survival probability for 1 year, 2 years, or 3 years caategories [42]. The sixth model by Zhao et al. predicts 6-month, or 12-month survival based on therapy form, resection status, KPS, and age [43]. Different from the example presented for the biopsy decision of prostate cancer, in this scenario, all base models output survival estimates, but vary with regards to their output details (days, months, years). The fusion model has the task of combining them in a meaningful way. A

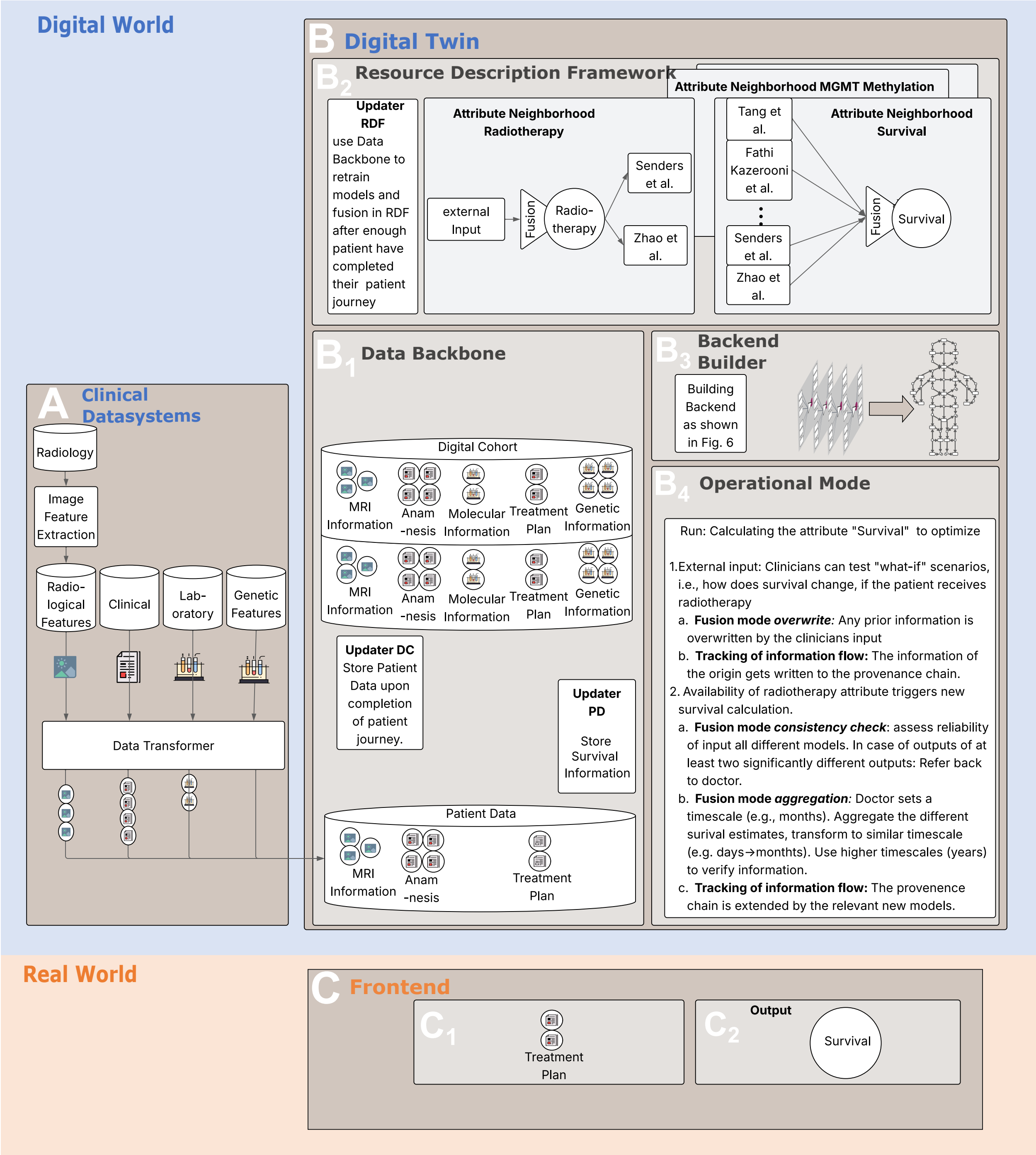


Figure 4: Schematic overview of our proposed software design for patient-centred DTs in glioblastoma survival estimation. The figure is similar to Fig. 2 of the main text, showing Clinical Datasystems (A), the DT itself (B), consisting of *data backbone* ($B_1$), the *Resource Description Framework* ($B_2$), which stores all available information about models and their links with attributes, which the *back-end builder* ($B_3$) uses to construct a knowledge graph upon which the *operational mode* ($B_4$) is performing predictions. The user interacts with the *front-end* (C).

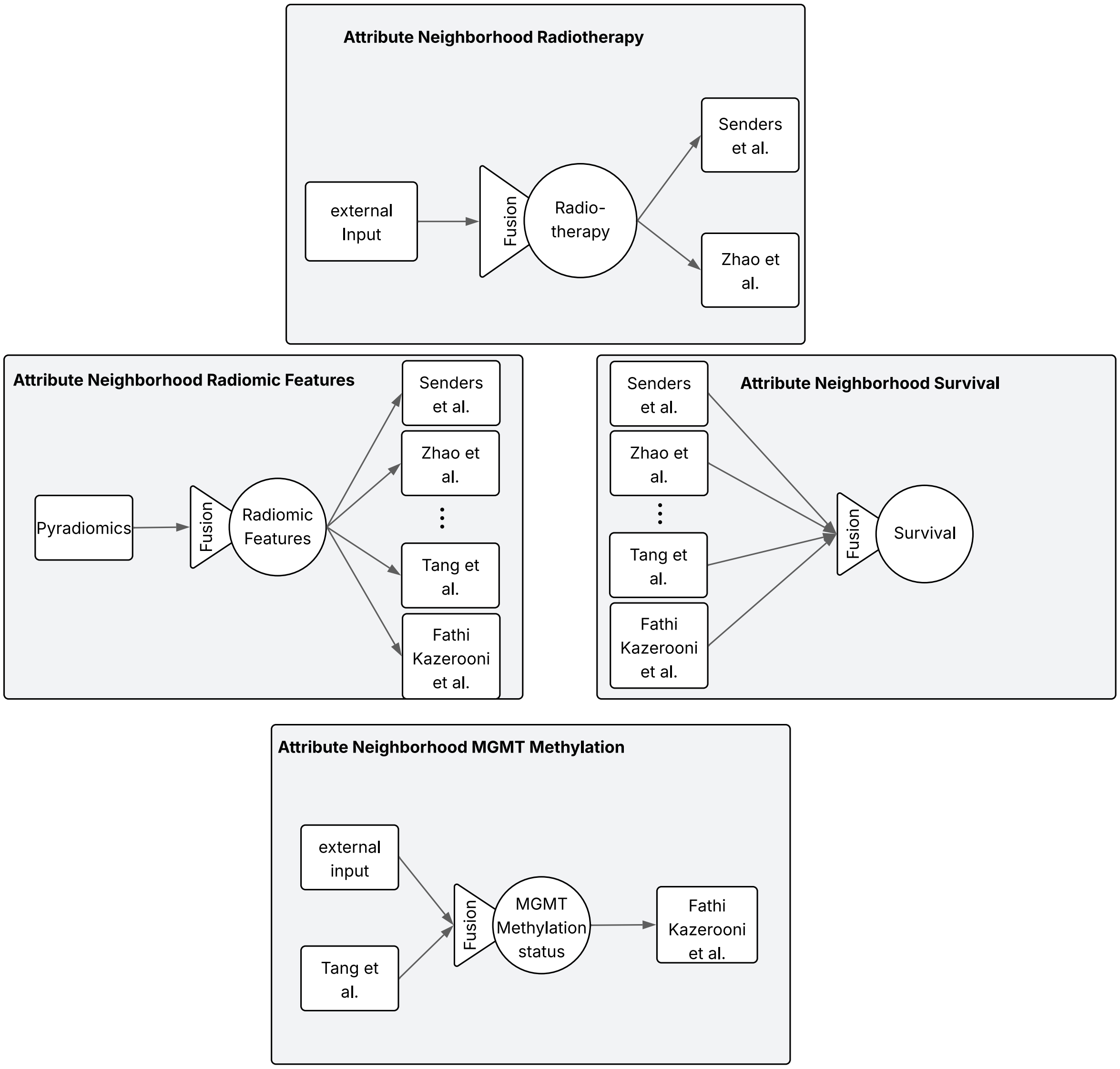


Figure 5: Selection of *attribute neighborhoods* for survival prediction in glioblastomas. On top, we have the "Attribute Neighborhood Radiotherapy" attribute. This is the starting point. Radiotherapy information will be passed to two models, namely Senders et al. and Zhao et al. Both models are also in the "Attribute Neighborhood Radiomic Features". As we assume this information to be present, this feature can inform models of Tang et al. and Fathi Kazerooni et al. At the bottom, the "Attribute Neighborhood MGMT Methylation" shows, that both models, Tang et al. and Fathi Kazerooni et al. are connected via the MGMT methylation status. At the right side, the "Attribute Neighborhood Survival" is shown. It collects all models that estimate the survival of the patient. For clarity, only the four models also shown in the other attribute neighborhoods are visualized. The connected graph is then shown in Fig. 6

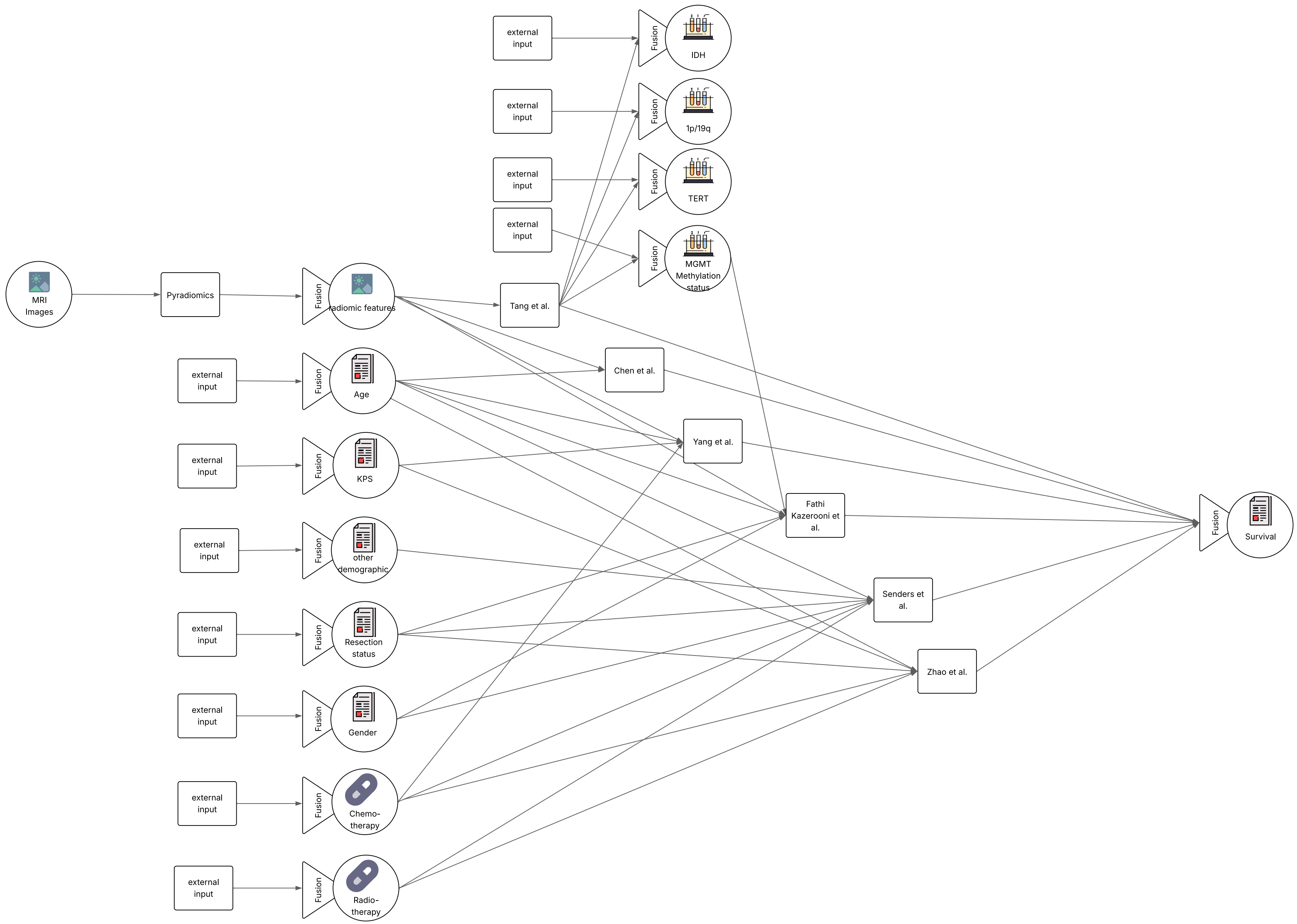


Figure 6: Detailed representation of the knowledge graph for "Patient Sarah Jane". The knowledge graph represents the interrelations of the models and data from imaging to survival prediction as constructed by the Backend Builder (B3).

possible mechanism of the fusion model will be explained later.

For the *backend builder* ($B_3$) to build the *knowledge graph*, it has to combine the most relevant attribute neighborhoods, which are visualized in Fig. 5, including the "Attribute Neighborhood Radiotherapy", "Attribute Neighborhood Radiomic Features", "Attribute Neighborhood MGMT Methylation" and "Attribute Neighborhood Survival". As our patient has imaging information, we assume radiomic features are available for the Digital Twin. The radiomic features inform four models, namely Sanders et al., Zhao et al., Tang et al., and Fahi Kazerooni et al.. The model of Tang et al. not only predicts survival, but also genetic information from the MRI images, radiomic features, and clinical features. Specifically, the model predicts the MGMT methylation status, which, on the other hand, is an input for the model of Fathi Kazerooni et al.. Thus, the "MGMT methylation status" adds a connection between those two models in the knowledge graph. Although given such cross-connections, the complete graph will be more complex, the local structure will always be a simple coupling of different *base models* via data attributes and their fusion models, which defines the *attribute neighborhoods*. Figure 6 visualizes the overall knowledge graph structure, including all additionally required patient attributes for the base models and their relations. From left to right, we show the input data, then an intermediate layer, and finally our desired outcome attribute. The input layer is the available clinical data. The intermediate layer represents the cross-connection of the models of Tang et al. and Fathi Kazerooni et al., as both models are connected via an output-input relation, as explained before. At the far right end, all models inform the survival attribute and the corresponding fusion model.

The *operational mode* controls the propagation and aggregation of information in the knowledge graph. Information from the MRI Images is extracted as radiomic features. The radiomic features are used by the models of Yang et al., Senders et al., and Zhao et al., which compute their survival estimate based on the given therapeutic approach. Additionally, Tang et al. use radiomic features together with the original images, age, and gender data to predict a survival estimate, MGMT methylation status and other mutation information. Although this model is independent of the treatment plan, we can envision such models existing. Such models can easily be added to the knowledge graph in the future. The *fusion model* for the MGMT methylation status has to combine a model output and an external input. Therefore, it is in an *overwrite mode*, similar to our previous example for biopsy decision. If present, the fusion always prefers the external input and discards the model output.

The remaining model of Fathi Kazerooni et al. has now all the needed input features and calculates its survival estimate. Now, all models have predicted the survival, but at different levels of detail (days, months, years). The fusion now has the task of combining all this information for the clinician into one best estimate of survival. This is the aggregation mode. Unlike a simple combination, the fusion has to transform all outputs so that they have the same meaning. For survival, this is the timescale. We assume this to be externally set by the doctor, e.g., he/she wants to know the chance for a 6-month survival. In this case, the fusion would use all models that provide the survival probability at this level of detail or higher level of detail, e.g., survival probability in days. The information with higher level of detail would be integrated to represent the wanted information, thus integrating from 0 to 180 days. Any information with a lower level of detail, e.g., survival probability per year, would be used as verification, e.g., by ensuring the 1-year survival estimate should not exceed the 6-month survival estimate. If the verification fails, all information should be passed back to the doctor, and conflicts should be highlighted in an interactive output scheme. Then the information flow gets tracked by adding all models except the verification models to the provenance chain $\mathcal{P}$ of the survival attribute.

Finally, the *updater PD* updates the Patient Data with the recent best estimates of survival. After the real survival of the patient is known, typically in the order of months to years, the *updater DC* adds all measured information to the *digital cohort*. After an even longer time period, the *updater RDF* uses the past cases for retraining the individual base and fusion models in the knowledge graph. This timer period is in the order of years, but it depends on other factors, like the number of patients collected at the clinic and the heterogeneity of the clinical patient journey, and therefore, the availability and missingness of specific data.

By giving the best possible survival estimate for our patient dependent on a therapy, the clinician is now able to choose the best treatment plan.

## References

[1] Steven M. Schwartz, Kevin Wildenhaus, Amy Bucher, and Brigid Byrd. Digital Twins and the Emerging Science of Self: Implications for Digital Health Experience Design and “Small” Data. *Frontiers in Computer Science*, 2, 2020.

[2] Christopher J. Kelly. Key challenges for delivering clinical impact with artificial intelligence. *BMC Medicine*, 17(1):1–9, October 2019.

[3] Michael G. Kapteyn, Jacob V. R. Pretorius, and Karen E. Willcox. A probabilistic graphical model foundation for enabling predictive digital twins at scale. *Nature Computational Science*, 1(5):337–347, May 2021.

[4] Sun Sun, Erik Stenberg, Lars Lindholm, Klas-Göran Salén, Karl A. Franklin, Nan Luo, and Yang Cao. Prediction of quality-adjusted life years (QALYs) after bariatric surgery using regularized linear regression models: results from a Swedish nationwide quality

register. *Obesity Surgery*, 33(8):2452–2462, August 2023.

[5] Claudio Capelli, Emilie Sauvage, Giuliano Giusti, Giorgia M. Bosi, Hopewell Ntsinjana, Mario Carminati, Graham Derrick, Jan Marek, Sachin Khambadkone, Andrew M. Taylor, and Silvia Schievano. Patient-specific simulations for planning treatment in congenital heart disease. *Interface Focus*, 8(1):20170021, February 2018.

[6] Wade P. Smith, Minsun Kim, Clay Holdsworth, Jay Liao, and Mark H. Phillips. Personalized treatment planning with a model of radiation therapy outcomes for use in multiobjective optimization of IMRT plans for prostate cancer. *Radiation Oncology*, 11(1):38, December 2016.

[7] Thanveer Shaik, Xiaohui Tao, Niall Higgins, Lin Li, Raj Gururajan, Xujuan Zhou, and U. Rajendra Acharya. Remote patient monitoring using artificial intelligence: Current state, applications, and challenges. *WIREs Data Mining and Knowledge Discovery*, 13(2):e1485, 2023.

[8] Katharine E. Henry, David N. Hager, Peter J. Pronovost, and Suchi Saria. A targeted real-time early warning score (trewscore) for septic shock. *Science Translational Medicine*, 7(299):299ra122–299ra122, 2015.

[9] Jing Xia, Su Pan, Min Zhu, Guolong Cai, Molei Yan, Qun Su, Jing Yan, and Gangmin Ning. A long short-term memory ensemble approach for improving the outcome prediction in intensive care unit. 2019(1):8152713, 2019.

[10] Marcus Eng Hock Ong, Christina Hui Lee Ng, Ken Goh, Nan Liu, Zhi Xiong Koh, Nur Shahidah, Tong Tong Zhang, Stephanie Fook-Chong, and Zhiping Lin. Prediction of cardiac arrest in critically ill patients presenting to the emergency department using a machine learning score incorporating heart rate variability compared with the modified early warning score. *Critical Care*, 16(3):R108, 2012.

[11] Jared M. Campbell, Elspeth Raymond, Michael E. O'Callaghan, Andrew D. Vincent, Kerri R. Beckmann, David Roder, Sue Evans, John McNeil, Jeremy Millar, John Zalcberg, Martin Borg, and Kim L. Moretti. Optimum Tools for Predicting Clinical Outcomes in Prostate Cancer Patients Undergoing Radical Prostatectomy: A Systematic Review of Prognostic Accuracy and Validity. *Clinical Genitourinary Cancer*, 15(5):e827–e834, October 2017.

[12] Hasna El Haji, Amine Souadka, Bhavik N. Patel, Nada Sbihi, Gokul Ramasamy, Bhavika K. Patel, Mounir Ghogho, and Imon Banerjee. Evolution of Breast Cancer Recurrence Risk Prediction: A Systematic Review of Statistical and Machine Learning-Based Models. *JCO clinical cancer informatics*, 7:e2300049, August 2023.

[13] Farah Shamout, Tingting Zhu, and David A. Clifton. Machine learning for clinical outcome prediction. *IEEE Reviews in Biomedical Engineering*, 14:116–126, 2021.

[14] J. Masison, J. Beezley, Y. Mei, Hal Ribeiro, A. C. Knapp, L. Sordo Vieira, B. Adhikari, Y. Scindia, M. Grauer, B. Helba, W. Schroeder, B. Mehrad, and R. Laubenbacher. A modular computational framework for medical digital twins. *Proceedings of the National Academy of Sciences*, 118(20):e2024287118, May 2021.

[15] Zitao Liu and Milos Hauskrecht. Clinical time series prediction: Toward a hierarchical dynamical system framework. *Artificial Intelligence in Medicine*, 65(1):5–18, September 2015.

[16] Zitao Liu and Milos Hauskrecht. A Personalized Predictive Framework for Multivariate Clinical Time Series via Adaptive Model Selection. In *Proceedings of the 2017 ACM on Conference on Information and Knowledge Management*, pages 1169–1177, Singapore Singapore, November 2017. ACM.

[17] J. Geoffrey Chase, Jean-Charles Preiser, Jennifer L. Dickson, Antoine Pironet, Yeong Shiong Chiew, Christopher G. Pretty, Geoffrey M. Shaw, Balazs Benyo, Knut Moeller, Soroush Safaei, Merryn Tawhai, Peter Hunter, and Thomas Desaive. Next-generation, personalised, model-based critical care medicine: a state-of-the art review of in silico virtual patient models, methods, and cohorts, and how to validation them. *BioMedical Engineering OnLine*, 17(1):24, December 2018.

[18] Dessislava Petrova-Antonova, Ivaylo Spasov, Iva Krasteva, Ilina Manova, and Sylvia Ilieva. A Digital Twin Platform for Diagnostics and Rehabilitation of Multiple Sclerosis. In Osvaldo Gervasi, Beniamino Murgante, Sanjay Misra, Chiara Garau, Ivan Blečić, David Taniar, Bernady O. Apduhan, Ana Maria A.C. Rocha, Eufemia Tarantino, Carmelo Maria Torre, and Yeliz Karaca, editors, *Computational Science and Its Applications – ICCSA 2020*, pages 503–518, Cham, 2020. Springer International Publishing.

[19] Yifeng Li, Fang-Xiang Wu, and Alioune Ngom. A review on machine learning principles for multi-view biological data integration. *Briefings in Bioinformatics*, 19(2):325–340, March 2018.

[20] Carlos A. Brandl, Anna-Katharina Nitschke, Fabian Egersdörfer, and Matthias Weidemüller. A personalized and evidence-based clinical decision support system using ensemble learning. In *2025 47th Annual International Conference of the IEEE Engineering in Medicine & Biology Society (EMBC)*, 2025. Submission accepted. To be published.

[21] Seniha Esen Yuksel, Joseph N. Wilson, and Paul D. Gader. Twenty Years of Mixture of Experts. *IEEE Transactions on Neural Networks and Learning Systems*, 23(8):1177–1193, August 2012.

[22] Simon S. Haykin. *Neural Networks: A Comprehensive Foundation*. Prentice Hall, Upper Saddle River, N.J, 2nd ed edition, 1999.

[23] Jorge Corral-Acero, Francesca Margara, Maciej Marciniak, Cristobal Rodero, Filip Loncaric, Yingjing Feng, Andrew Gilbert, Joao F Fernandes, Hassaan A Bukhari, Ali Wajdan, Manuel Villegas Martinez, Mariana Sousa Santos, Mehrdad Shamohammdi, Hongxing Luo, Philip Westphal, Paul Leeson, Paolo DiAchille, Viatcheslav Gurev, Manuel Mayr, Liesbet Geris, Pras Pathmanathan, Tina Morrison, Richard Cornelussen, Frits Prinzen, Tammo Delhaas, Ada Doltra, Marta Sitges, Edward J Vigmond, Ernesto Zacur, Vicente Grau, Blanca Rodriguez, Espen W Remme, Steven Niederer, Peter Mortier, Kristin McLeod, Mark Potse, Esther Pueyo, Alfonso Bueno-Orovio, and Pablo Lamata. The 'Digital Twin' to enable the vision of precision cardiology. *European Heart Journal*, 41(48):4556–4564, December 2020.

[24] Ian A. Scott. Machine Learning and Evidence-Based Medicine. *Annals of Internal Medicine*, 169(1):44, July 2018.

[25] Ahmad A. Abujaber, Abdulqadir J. Nashwan, and Adam Fadlalla. Harnessing machine learning to support evidence-based medicine: A pragmatic reconciliation framework. *Intelligence-Based Medicine*, 6:100048, 2022.

[26] Alejandro Barredo Arrieta, Natalia Díaz-Rodríguez, Javier Del Ser, Adrien Bennetot, Siham Tabik, Alberto Barbado, Salvador Garcia, Sergio Gil-Lopez, Daniel Molina, Richard Benjamins, Raja Chatila, and Francisco Herrera. Explainable artificial intelligence (XAI): Concepts, taxonomies, opportunities and challenges toward responsible AI. 58:82–115.

[27] Aya Abdelsalam Ismail, Sercan Ö Arik, Jinsung Yoon, Ankur Taly, Soheil Feizi, and Tomas Pfister. Interpretable Mixture of Experts for Structured Data, June 2022.

[28] Christoph Molnar. *Interpretable Machine Learning*. Lulu.com, 2019.

[29] Scott M Lundberg and Su-In Lee. A Unified Approach to Interpreting Model Predictions. In *Advances in Neural Information Processing Systems*, volume 30. Curran Associates, Inc., 2017.

[30] Andrea (AZQ) Haring. *S3-Leitlinie Prostatakarzinom*. AWMF, 2021.

[31] Derek J Van Booven, Manish Kuchakulla, Raghav Pai, Fabio S Frech, Reshna Ramasahayam, Pritika Reddy, Madhumita Parmar, Ranjith Ramasamy, and Himanshu Arora. A Systematic Review of Artificial Intelligence in Prostate Cancer. *Research and Reports in Urology*, 13:31–39, January 2021.

[32] Amir Torab-Miandoab, Taha Samad-Soltani, Ahmadreza Jodati, and Peyman Rezaei-Hachesu. Interoperability of heterogeneous health information systems: a systematic literature review. *BMC Medical Informatics and Decision Making*, 23(1), January 2023.

[33] Guodong Jing, Pengyi Xing, Zhihui Li, Xiaolu Ma, Haidi Lu, Chengwei Shao, Yong Lu, Jianping Lu, and Fu Shen. Prediction of clinically significant prostate cancer with a multimodal MRI-based radiomics nomogram. 12. Publisher: Frontiers.

[34] Jan Philipp Radtke, Manuel Wiesenfarth, Claudia Kesch, Martin T. Freitag, Celine D. Alt, Kamil Celik, Florian Distler, Wilfried Roth, Kathrin Wieczorek, Christian Stock, Stefan Duensing, Matthias C. Roethke, Dogu Teber, Heinz-Peter Schlemmer, Markus Hohenfellner, David Bonekamp, and Boris A. Hadaschik. Combined clinical parameters and multiparametric magnetic resonance imaging for advanced risk modeling of prostate cancer—patient-tailored risk stratification can reduce unnecessary biopsies. 72(6):888–896.

[35] Donna P. Ankerst, Johanna Straubinger, Katharina Selig, Lourdes Guerrios, Amanda De Hoedt, Javier Hernandez, Michael A. Liss, Robin J. Leach, Stephen J. Freedland, Michael W. Kattan, Robert Nam, Alexander Haese, Francesco Montorsi, Stephen A. Boorjian, Matthew R. Cooperberg, Cedric Poyet, Emily Vertosick, and Andrew J. Vickers. A Contemporary Prostate Biopsy Risk Calculator Based on Multiple Heterogeneous Cohorts. *European Urology*, 74(2):197–203, August 2018.

[36] Roya Poursaeed, Mohsen Mohammadzadeh, and Ali Asghar Safaei. Survival prediction of glioblastoma patients using machine learning and deep learning: a systematic review. 24(1):1581.

[37] Michael Weller, Martin van den Bent, Matthias Preusser, Emilie Le Rhun, Jörg C. Tonn, Giuseppe Minniti, Martin Bendszus, Carmen Balana, Olivier Chinot, Linda Dirven, Pim French, Monika E. Hegi, Asgeir S. Jakola, Michael Platten, Patrick Roth, Roberta Rudà, Susan Short, Marion Smits, Martin J. B. Taphoorn, Andreas von Deimling, Manfred Westphal, Riccardo Soffietti, Guido Reifenberger, and Wolfgang Wick. EANO guidelines on the diagnosis and treatment of diffuse gliomas of adulthood. *Nature Reviews Clinical Oncology*, 18(3):170–186, March 2021.

[38] Hao Chen, Yang Liu, Xiaoying Pan, Qing Yang, Yongqian Qiang, and X Sharon Qi. A subregion-based survival prediction framework for GBM via multi-sequence MRI space optimization and clustering-based feature bundling and construction. 68(12):125005. Publisher: IOP Publishing.

[39] Anahita Fathi Kazerooni, Sanjay Saxena, Erik Toorens, Danni Tu, Vishnu Bashyam, Hamed Akbari, Elizabeth Mamourian, Chiharu Sako, Costas Koumenis, Ioannis Verginadis, Ragini Verma, Russell T. Shinohara, Arati S. Desai, Robert A. Lustig, Steven Brem, Suyash Mohan, Stephen J. Bagley, Tapan

Ganguly, Donald M. O'Rourke, Spyridon Bakas, MacLean P. Nasrallah, and Christos Davatzikos. Clinical measures, radiomics, and genomics offer synergistic value in AI-based prediction of overall survival in patients with glioblastoma. 12(1):8784. Publisher: Nature Publishing Group.

[40] Yang Yang, Yu Han, Shijie Zhao, Gang Xiao, Lei Guo, Xin Zhang, and Guangbin Cui. Spatial heterogeneity of edema region uncovers survival-relevant habitat of glioblastoma. 154:110423.

[41] Zhenyu Tang, Yuyun Xu, Lei Jin, Abudumijiti Aibaidula, Junfeng Lu, Zhicheng Jiao, Jinsong Wu, Han Zhang, and Dinggang Shen. Deep learning of imaging phenotype and genotype for predicting overall survival time of glioblastoma patients. 39(6):2100–2109.

[42] Joeky T. Senders, Patrick Staples, Alireza Mehrtash, David J. Cote, Martin J. B. Taphoorn, David A. Reardon, William B. Gormley, Timothy R. Smith, Marike L. Broekman, and Omar Arnaout. An online calculator for the prediction of survival in glioblastoma patients using classical statistics and machine learning. 86(2):E184.

[43] Rachel Zhao, Jonathan Zeng, Kimberly DeVries, Ryan Proulx, and Andra Valentina Krauze. Optimizing management of the elderly patient with glioblastoma: Survival prediction online tool based on BC cancer registry real-world data. 4(1):vdac052.
